# A non-transmissible live attenuated SARS-CoV-2 vaccine

**DOI:** 10.1016/j.ymthe.2023.05.004

**Published:** 2023-05-26

**Authors:** Julia M. Adler, Ricardo Martin Vidal, Anne Voß, Sandra Kunder, Mariana Nascimento, Azza Abdelgawad, Christine Langner, Daria Vladimirova, Nikolaus Osterrieder, Achim D. Gruber, Dusan Kunec, Jakob Trimpert

**Affiliations:** 1 Institut für Virologie, Freie Universität Berlin, 14163 Berlin, Germany; 2 Institut für Tierpathologie, Freie Universität Berlin, 14163 Berlin, Germany

**Keywords:** SARS-CoV-2, COVID-19, vaccine, virus transmission, live attenuated virus, pneumonia, mucosal vaccination

## Abstract

Live attenuated vaccines (LAVs) administered via the mucosal route may offer better control of the COVID-19 pandemic than non-replicating vaccines injected intramuscularly. Conceptionally, LAVs have several advantages, including presentation of the entire antigenic repertoire of the virus, and the induction of strong mucosal immunity. Thus, immunity induced by LAV could offer superior protection against future surges of COVID-19 cases caused by emerging SARS-CoV-2 variants. However, LAVs carry the risk of unintentional transmission. To address this issue, we investigated whether transmission of a SARS-CoV-2 LAV candidate can be blocked by removing the furin cleavage site (FCS) from the spike protein. The level of protection and immunity induced by the attenuated virus with the intact FCS was virtually identical to the one induced by the attenuated virus lacking the FCS. Most importantly, removal of the FCS completely abolished horizontal transmission of vaccine virus between cohoused hamsters. Furthermore, the vaccine was safe in immunosuppressed animals and showed no tendency to recombine *in vitro* or *in vivo* with a SARS-CoV-2 field strain. These results indicate that removal of the FCS from SARS-CoV-2 LAV is a promising strategy to increase vaccine safety and prevent vaccine transmission without compromising vaccine efficacy.

## Graphical abstract

**Figure:**
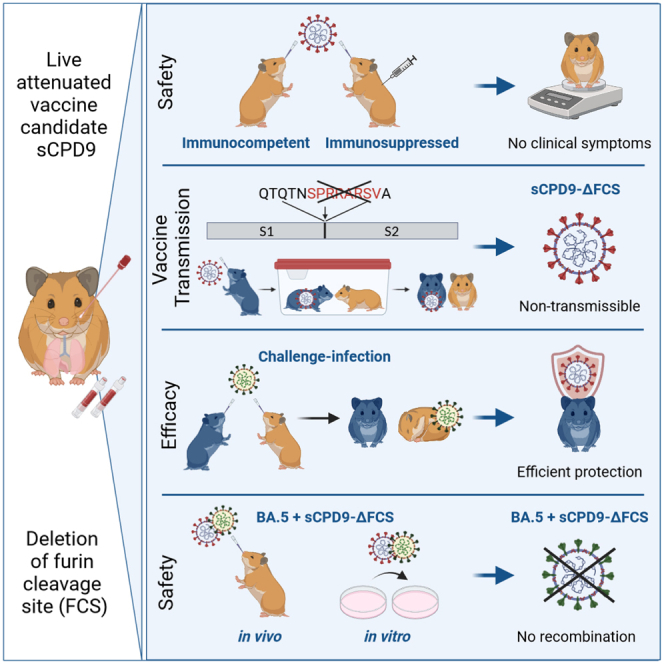

## Teaser

Adler and colleagues show that removing the furin cleavage site from live attenuated vaccine candidate sCPD9 blocked horizontal transmission between hamsters without compromising safety or efficacy. The updated vaccine was safe for immunocompromised hamsters and did not show tendency to recombine with field strains *in vitro* or *in vivo*.

## Introduction

Unintentional spread of vaccine viruses from vaccinated to unvaccinated individuals can complicate the use of transmissible live attenuated vaccines (LAVs).^1^^,^^2^^,^^3^^,^^4^ While self-dissemination is desirable in some scenarios, specifically when herd immunity is sought in wildlife,^5^ uncontrolled circulation of vaccine viruses potentially increases the risk of reversion to virulence.^2^^,^^3^^,^^4^ Recombination between different vaccine viruses or vaccine and field viruses is particularly problematic because it can give rise to recombinants with increased virulence, transmissibility, or immune evasion capabilities.^6^^,^^7^^,^^8^^,^^9^ The rapid evolution of SARS-CoV-2 urges extra caution in the use of LAVs with respect to their potentially irrevocable circulation. Moreover, transmission of attenuated viruses to immunocompromised individuals is a danger inherent to the use of transmissible LAVs.^10^

In response to the COVID-19 pandemic, we developed a series of live attenuated SARS-CoV-2 vaccine candidates through large-scale recoding of the SARS-CoV-2 genome using an approach called codon pair deoptimization (CPD), also known as synthetic attenuated virus engineering.^11^ This innovative approach involves construction of viruses with genomes in which specific parts have been recoded *in silico*. The goal of this process is to create a large number of codon pairs that are rarely used in the host organism, which reduces protein production from the recoded genes and leads to attenuation of the mutant viruses.^12^ It is important to note that CPD only exchanges the synonymous codons in recoded sequences, meaning that the recoded viruses retain the same antigens as the pathogenic parent. This antigenic identity, coupled with the replicative potential of the virus, enables the attenuated virus to fully engage the immune system of the host and stimulate robust immune responses.^12^^,^^13^^,^^14^ CPD has been successfully used to attenuate a variety of RNA^12^^,^^13^ and DNA viruses,^14^^,^^15^ making it a rapid and efficient method for vaccine development.

In our previous studies, we have evaluated live attenuated SARS-CoV-2 candidates and found that our leading LAV candidate, sCPD9, was highly attenuated, induced robust immune responses, and protected Syrian and Roborovski hamsters against a challenge with the ancestral SARS-CoV-2 variant B.1, as well as subsequent variants B.1.1.7 (Alpha), B.1.351 (Beta), and B.1.617.2 (Delta) that emerged during the pandemic.^11^^,^^16^^,^^17^ Most importantly, sCPD9 outperformed intramuscularly administered adenoviral-vectored and mRNA vaccines in its ability to induce systemic and mucosal immunity.^17^ Owing to its excellent safety profile, sCPD9 was recently downgraded to biosafety-level (BSL) 2 settings, which is an important precedent facilitating vaccine production for clinical trials.^18^ In the current study, we examined the transmission of SARS-CoV-2 LAV candidate sCPD9, and suggest a simple solution to prevent the transmission of SARS-CoV-2 LAV more generally.

Entry of SARS-CoV-2 into host cells is mediated by its major surface protein, spike glycoprotein (S). The trimeric type I fusion protein has two subunits. The N-terminal subunit S1 initiates infection by binding to its cellular receptor, angiotensin-converting enzyme 2 (ACE2), while the C-terminal S2 subunit mediates fusion between the viral and host cell membranes. To enable cell entry, S must be proteolytically activated by host proteases. Activation involves proteolytic cleavage of S at the S1/S2 boundary, at two sites termed S1/S2 and S2′. The priming cleavage at the S1/S2 site generates subunits S1 and S2, which are held together by non-covalent interactions. This step causes conformational changes that allow the S1 subunit to bind to ACE2 via its receptor binding domain.^19^ Unlike other closely related viruses, SARS-CoV-2 contains a unique polybasic cleavage motif (PRRAR685↓) at the S1/S2 site, also referred to as the furin cleavage site (FCS) because it is primarily cleaved by furin and other furin-like proteases.^20^ Cleavage at the S1/S2 site occurs during S biogenesis and approximately 50% of S is primed.^21^ Although cleavage at the S1/S2 site is not essential for virus entry, it enhances subsequent cleavage at the S2′ site.^21^ On the other hand, cleavage at the S2′ site is essential for virus entry, because it exposes the hydrophobic fusion peptide, which then mediates fusion between viral and host membranes. The S2′ cleavage determines the entry route of SARS-CoV-2. When the host cell expresses TMPRSS2, the virus is activated at the cell surface and rapidly enters the cells via cell fusion in a pH-independent manner.^22^ In contrast, if TMPRSS2 or related proteases are absent, the virus is endocytosed and virus entry is mediated at lower pH by endosomal cysteine proteases cathepsin L or B.^19^^,^^22^

To prevent transmission of the vaccine virus sCPD9, we deleted the FCS from its spike protein. Previous studies showed that removal of the FCS renders mutant viruses non-transmissible and strongly attenuated.^23^^,^^24^^,^^25^^,^^26^ However, since removal of the FCS can enhance viral attenuation, combining the modification with additional attenuating mutation(s) entails the risk of an overly attenuated virus, which may in turn compromise immune responses and protection induced by vaccination. Therefore, it is important to compare transmissibility and protective efficacy of LAV candidates that lack the FCS with those that contain an intact FCS. On the other hand, if removal of the FCS does not compromise the protective efficacy of LAV candidates, then its removal is desirable because it would increase the safety of LAV candidates. In addition, the introduction of an additional and independent attenuating mutation into the virus genome further reduces the likelihood that LAV candidates will revert to a pathogenic phenotype.

Furthermore, aside from eliminating transmission and increasing vaccine safety, removing the FCS has an important practical advantage for large-scale production of SARS-CoV-2 LAV. When propagated in cells that do not express TMPRSS2, such as Vero E6 cells, which are commonly used for this purpose, virus variants lacking functional FCS become rapidly dominant because they outcompete variants with intact FCS.^24^^,^^27^^,^^28^^,^^29^^,^^30^^,^^31^^,^^32^ Consistent with these reports, we found that sCPD9 also rapidly loses its FCS when propagated in cells that do not express TMPRSS2.^18^ Thus, removal of the FCS would increase the genetic stability of vaccine viruses during production, and may result in higher virus titers on TMPRSS2-deficient cell lines.

## Results

### Deletion of the FCS accelerates replication of sCPD9

To reduce the transmissibility and increase the genetic stability of sCPD9, we generated sCPD9-ΔFCS, a derivative of sCPD9, which features a deletion of the FCS in the S glycoprotein. We engineered the sCPD9-ΔFCS mutant to contain the “Bristol deletion,” which emerged during serial passage on cultured Vero E6 cells.^32^ The introduced deletion is 24 nucleotides long, resulting in the removal of eight amino acids (SPRRARSV) from S.^32^

**Figure 1:**
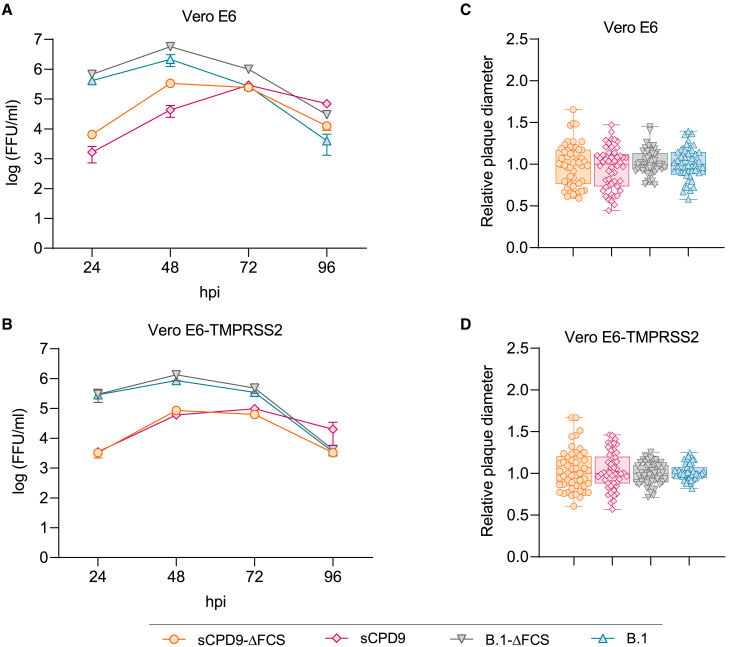
Multi-step growth kinetics and plaque sizes (A, B) Multi-step growth kinetics of sCPD9-ΔFCS, sCPD9, B.1-ΔFCS or B.1 viruses on Vero E6 (A) and Vero E6-TMPRSS2 (B) cells. Confluent cells grown in T25 flasks were infected with 100 ffu of the indicated virus and viral titers were determined 24, 48, 72, and 96 h post infection (hpi). The results are shown as means ± SD of duplicates. (C, D) Plaque size diameter of sCPD9-ΔFCS, sCPD9, B.1-ΔFCS, or B.1 viruses on Vero E6 (C) and Vero E6-TMPRSS2 (D) cells. The box-plots relative plaque diameters of 50 plaques normalized against the average plaque diameter of the sCPD9-ΔFCS virus. Shown are the mean and 25th to 75th percentiles with whiskers (min to max).

Several studies have shown that SARS-CoV-2 variants lacking the FCS have a distinct growth advantage over viruses with an intact FCS on different Vero cell lines. Our findings were in accordance with previously reported results, as we observed a trend toward faster virus growth in Vero E6 cells upon removal of the FCS (Figure 1A). However, sCPD9-ΔFCS showed a similar growth advantage compared to sCPD9 also on TMPRSS2-expressing Vero E6 cells (Figure 1B). No appreciable difference in the size of virus plaques was observed at 48 h post infection on either cell line (Figures 1C and 1D).

### Deletion of FCS prevents transmission of sCPD9-ΔFCS

**Figure 2:**
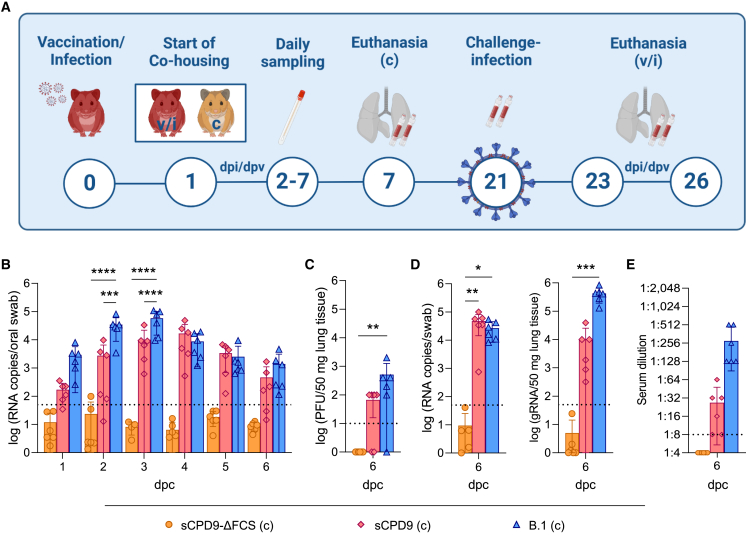
Virological findings in contact hamsters (A) Schematic overview of the experimental setup. Syrian hamsters were vaccinated either with sCPD9-ΔFCS or sCPD9, or infected with B.1 (wild type) on day 0. On day 1 after the vaccination/infection (dpv/dpi) naive contact hamsters (c) were placed in cohabitation with infected hamsters (i). Contact animals were sampled daily and euthanized after 6 days of cohabitation (dpc). The remaining infected hamsters were challenge-infected with SARS-CoV-2 variant Delta 21 days after vaccination/infection and euthanized on days 23 and 26. (B) Viral genomic RNA (gRNA) copies detected in daily oral swabs from contact hamsters n = 6 (1–6 dpc). Error bars show SD. Statistical analysis was performed using two-way ANOVA and Tukey’s multiple comparison tests. ∗p < 0.05, ∗∗p < 0.01, ∗∗∗p < 0.001, and ∗∗∗∗p < 0.0001. (C) Replication-competent virus particles in lung tissue. (D) gRNA copies detected in oropharyngeal swabs and homogenized lung tissue. (E) SARS-CoV-2 neutralization titers of sera collected at dpc 6 (upper detection limit = 1:1,024). (C–E). Error bars show SD. Dotted lines represent the limit of detection of each assay. Statistical analysis and p values were calculated using the Kruskal-Wallis test and Dunn’s multiple comparison test. ∗p < 0.05, ∗∗p < 0.01, and ∗∗∗p < 0.001.

**Figure 3:**
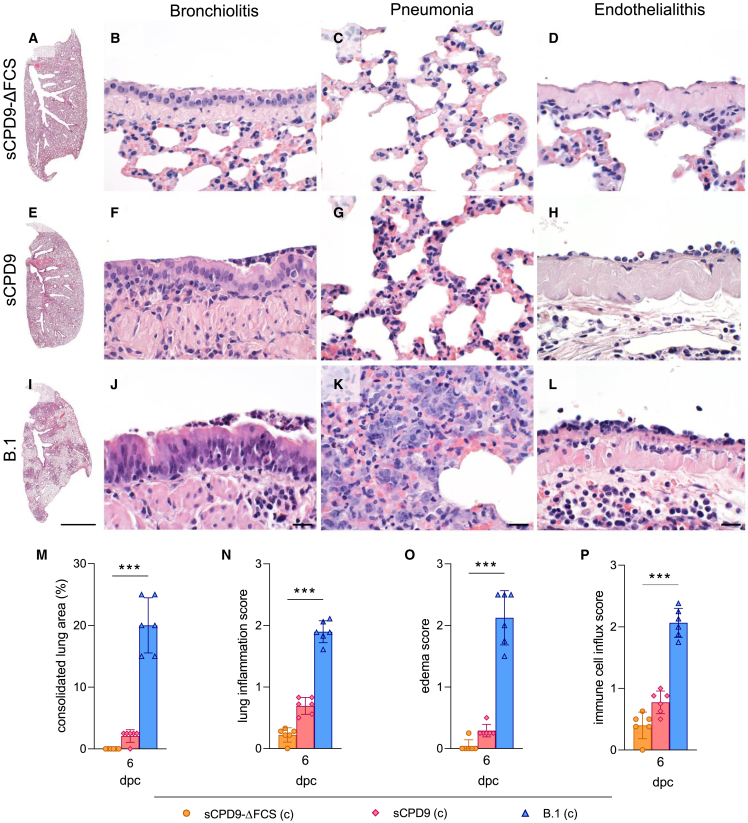
Histopathological findings in contact hamsters (A–L) Hematoxylin and eosin-stained sections of lungs of naive hamsters that were in contact with the sCPD9-ΔFCS-vaccinated (A)–(D), sCPD9-vaccinated (E)–(H), or B.1-infected hamsters (I)–(L) at day 6 post contact. The lungs of hamsters that were in contact with sCPD9-ΔFCS-vaccinated hamsters showed no signs of inflammation in whole lung scan of left lung lobes (A), major airways (B), peripheral lung tissue (C), or blood vessels (D) at dpi 6. The lungs of hamsters that were in contact with sCPD9-vaccinated hamsters developed hardly any consolidation in left lung lobe scans (E) and only mild bronchiolitis (F), interstitial pneumonia (G), and vascular endothelialitis (H). In contrast, the lungs of hamsters that had contact to B.1-infected hamsters had multifocal patchy consolidation of their lungs (I), marked suppurative and necrotizing to proliferative bronchiolitis (J), marked diffuse alveolar damage with hyperplastic alveolar epithelial cells (K), and strong vascular endothelialitis (L) at 6 dpc. Size bars, 1 cm (A, E, I) or 30 μm (all others). (M)–(P) Histopathological scoring of lung parameters (n = 6). (M) Consolidated lung area in percentage per group on 6 dpc. (N) Inflammatory damage of the lungs is semi-quantitatively assessed in the lung inflammation score including severity of pneumonia; influx of neutrophils, lymphocytes, and macrophages; bronchial epithelial necrosis; bronchitis; alveolar epithelial necrosis; perivascular lymphocyte cuffs; and pneumocyte type II hyperplasia. (O) Edema score accounts for perivascular and alveolar edema and (P) immune cell influx score includes infiltration of lymphocytes, neutrophils, and macrophages as well as perivascular lymphocyte cuffs. (M)–(P) Results are displayed in mean ± SD with symbols indicating individual values. Statistical analysis was done with Kruskal-Wallis and Dunn’s multiple comparisons tests. ∗p < 0.05, ∗∗p < 0.01, and ∗∗∗p < 0.001.

To investigate the ability of sCPD9 and sCPD9-ΔFCS to transmit to unvaccinated contact animals, we vaccinated six Syrian hamsters with sCPD9, sCPD9-ΔFCS, or infected with parental SARS-CoV-2 wild type (WT) (ancestral SARS-CoV-2 variant B.1).^33^ Twenty-four hours after infection, we cohoused each infected hamster with a naive, unvaccinated hamster in individually ventilated cages (Figure 2A). Oral swabs of all naive animals housed with WT-infected animals were strongly positive for SARS-CoV-2 RNA already after 1 day of cohabitation. All naive animals that were in contact with sCPD9-vaccinated subjects contracted the virus on days 1–3 of cohabitation and displayed a similar, although delayed, course of virus replication in the upper airways, compared with WT-infected animals. In contrast, naive animals that were in contact with sCPD9-ΔFCS-vaccinated hamsters did not test positive for SARS-CoV-2 RNA during the 6 days of cohousing with infected animals (Figures 2B–2D). Consistent with these results, seroconversion was detected at 6 days post contact (dpc) only in sera from naive animals that were cohoused with WT-infected and, to a lesser extent, sCPD9-vaccinated animals. Naive animals in contact with sCPD9-ΔFCS vaccinees remained seronegative until the endpoint of the study (Figure 2E). Natural transmission of WT SARS-CoV-2 caused expected COVID-19-like pneumonia in contact animals, as evidenced by histological examination on day 6 of cohabitation. Specifically, these hamsters developed marked patchy bronchointerstitial pneumonia with necrosuppurative bronchitis and bronchiolitis, diffuse alveolar damage, perivascular and alveolar edema, proliferation of alveolar type II epithelia, and vascular endotheliitis. In agreement with our previous findings, all inflammatory changes were greatly attenuated in animals that contracted sCPD9.^11^^,^^16^ Consistent with the negative virological results, lungs of animals cohabitated with the sCPD9-ΔFCS-vaccinated animals failed to show any evidence of pulmonary lesions (Figure 3).

### sCPD9-ΔFCS is highly attenuated in Syrian hamsters

**Figure 4:**
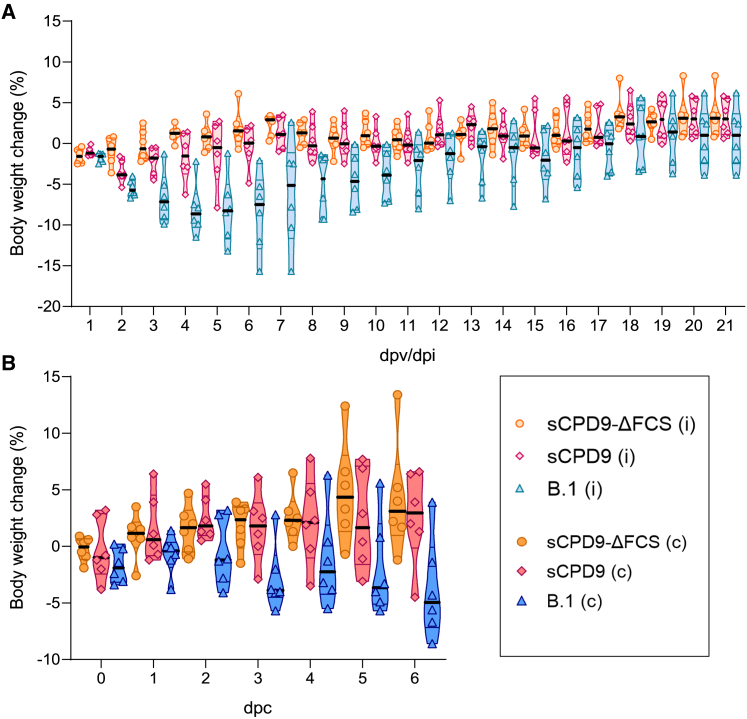
Change in body weight of experimental animals (A) Body weight of infected/vaccinated hamsters (i) during the first 21 days after infection/vaccination. (B) Body weight of contact animals (c) during the cohousing period. (A) and (B) Violin plots (truncated) show weights of individual animals (n = 6), group medians, and quartiles.

All hamsters remained clinically healthy after vaccination with sCPD9 or sCPD9-ΔFCS, whereas WT-infected animals showed expected moderate signs of disease, such as forced breathing and considerable weight loss during the first week after infection (Figure 4A). In the absence of other visible signs of disease, sCPD9-infected animals showed a trend toward slightly decreasing body weights, while sCPD9-ΔFCS-vaccinated animals presented with relatively stable body weights in the week after vaccination. Although all contact animals exposed to sCPD9-vaccinated or WT-infected animals contracted the respective virus, clinical signs of disease and body weight loss occurred only in animals that contracted WT virus (Figure 4B).

### Deletion of FCS does not reduce vaccine efficacy

**Figure 5:**
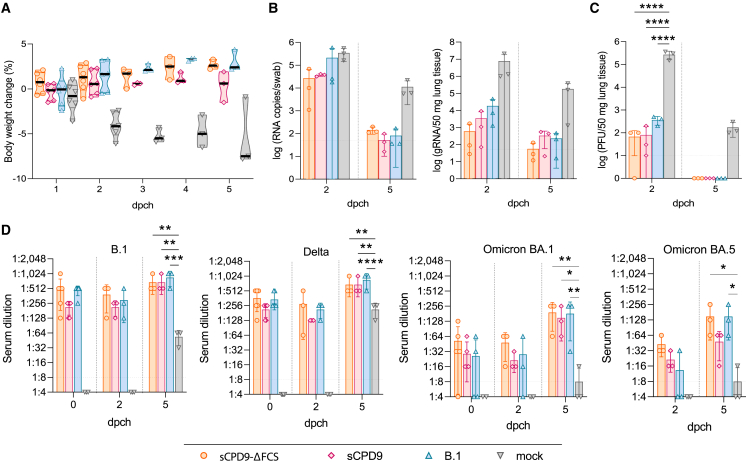
Clinical, virological, and serological results in hamsters after challenge infection with SARS-CoV-2 variant Delta (A) Change in body weight of animals after challenge infection. Violin plots (truncated) show weights of individual animals (n = 6), group medians, and quartiles. (B) Viral gRNA in oropharyngeal swabs and lung tissue. (C) Infectious virus particles in homogenized lung tissue (n = 3). (D) Neutralizing activity of hamster sera collected on day 0 (before challenge, n = 6), and on days 2 and 5 post challenge (dpch, n = 3) against SARS-CoV-2 variants B.1, Delta, and BA.1 and BA.5 (upper detection limit = 1:1,024). (B)–(D) Error bars display SD. Dotted lines show the lower limits of detection. The statistical analysis was performed using two-way ANOVA and Tukey’s multiple comparison tests. ∗p < 0.05, ∗∗p < 0.01, ∗∗∗p < 0.001, and ∗∗∗∗p < 0.0001.

All vaccinated or infected animals were challenged with the pathogenic SARS-CoV-2 Delta variant on day 21 after infection or vaccination. Vaccination or previous infection with WT did prevent overt signs of disease; none of the previously infected or vaccinated animals developed clinical signs of disease or exhibited the marked body weight loss observed in unvaccinated control animals (Figure 5A).

Protection against replication of the challenge virus was comparable in all three groups of animals. On day 2 after challenge, all groups showed high viral RNA loads in the upper respiratory tract (Figure 5B). However, these were reduced to levels near the detection limit by day 5 after challenge in all vaccinated or previously infected animals. Protection in the lower respiratory tract was more pronounced on day 2 after challenge infection with significantly lower viral RNA loads and minimal levels of replication-competent virus in the lung of infected animals (Figures 5B and 5C). By day 5 after challenge infection, RNA loads were near or below the detection limit, and no replicating virus was present in the lungs of any of the challenged subjects (Figures 5B and 5C).

We next compared humoral immune responses against SARS-CoV-2 WT, as well as the Delta variant used in challenge infection experiments and the more recent Omicron variants BA.1 and BA.5 (Figure 5D). The results show that vaccination/infection with sCPD9, sCPD9-ΔFCS, or WT viruses induced high and comparable levels of neutralizing antibodies prior to and following challenge infection. Importantly, neutralizing activity was detected across a range of four evolutionarily distant SARS-CoV-2 lineages. However, not surprisingly, serum neutralization was considerably weaker for BA.1 and BA.5, variants known for their prominent immune evasion properties. Overall, clinical, virological, and serological parameters confirmed the robust protection against challenge infection of both sCPD9 and sCPD9-ΔFCS vaccine candidates. Importantly, in all parameters measured, protection induced by vaccinations was comparable to protection conferred by infection with WT virus.

**Figure 6:**
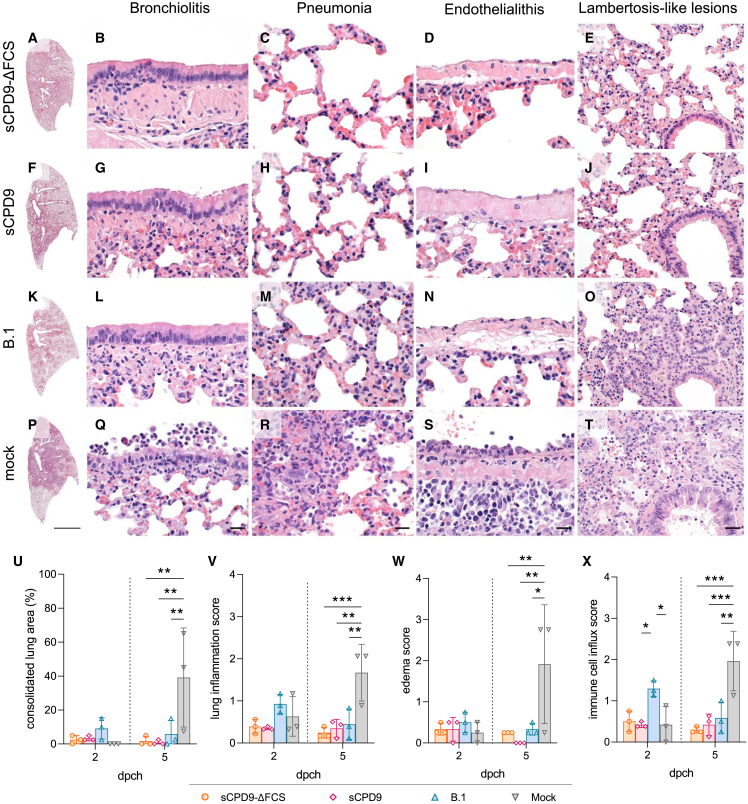
Pathological findings in hamsters after challenge infection with SARS-CoV-2 variant Delta The lungs of hamsters vaccinated with sCPD9-ΔFCS (A–E) or sCPD9 (F–J) had no or only minimal signs of pulmonary inflammation or other changes, including consolidation as seen in whole scans of left lung lobes (A, F), bronchiolitis (B, G), interstitial pneumonia (C, H), vascular endothelialitis (D, I), or lambertosis-like epithelial hyperplasia (E, J). Slightly stronger lesions were seen in hamsters that had been infected with B.1, including mild patchy consolidation of pulmonary parenchyma (K), moderate necrotizing and proliferative bronchiolitis (L), interstitial pneumonia (M), mild vascular endothelialitis (N), and marked lambertosis-like epithelial proliferation (O) 26 days after infection. In contrast to all three vaccinated/infected groups, unvaccinated (mock) hamsters developed strong histopathology lesions at 5 days after challenge, including marked patchy to confluent parenchymal consolidation (P), suppurative to necrotizing bronchiolitis (Q), marked diffuse alveolar damage (R), and strong vascular endothelialitis (S) but no lambertosis-like epithelial hyperplasia (T). (A) to (T), hematoxylin and eosin-stained sections of lesions representative of each group. Size bars, 1 cm (A, F, K, P), 50 μm (E, J, O, T), or 30 μm (all others). (U) Lung pathology scores with percentage of lung area consolidated by SARS-CoV-2 infection. (V) Lung inflammation score including influx of neutrophils, lymphocytes, and macrophages; bronchial epithelial necrosis; bronchitis; alveolar epithelial necrosis; and perivascular lymphocyte cuffs, as well as pneumocyte type II hyperplasia. (W) Pulmonary edema score including perivascular and alveolar edema and (X) immune cell influx score accounting for infiltration of lymphocytes, neutrophils, and macrophages, as well as perivascular lymphocyte cuffs (n = 3). (U–X) Results are shown in mean ± SD with symbols representing individual values. Data were analyzed using two-way ANOVA and Tukey’s multiple comparisons tests. ∗p < 0.05, ∗∗p < 0.01, and ∗∗∗p < 0.001.

Protective efficacies of sCPD9 and sCPD9-ΔFCS vaccine viruses were further determined by examining the lung histopathology of uninfected and sCPD9-, sCPD9-ΔFCS-vaccinated, or WT-infected hamsters on days 2 and 5 after challenge infection (Figure 6). On day 2, animals previously infected with WT virus showed a tendency toward increased influx of immune cells into major airways and respiratory parenchyma compared with animals that had been vaccinated with sCPD9 or sCPD9-ΔFCS. The situation was ameliorated by day 5 after challenge, suggesting that the potentially adverse reaction of previously WT-infected hamsters was transient and restricted to the time immediately following challenge infection, causing the pulmonary changes observed on day 2 after challenge infection. Importantly, all animals infected with WT or vaccinated with sCPD9 or sCPD9-ΔFCS showed excellent and largely similar protection against COVID-19-like pneumonia on day 5 after challenge. Only hamsters previously infected with WT virus showed slightly more immune cell infiltrates than the two vaccinated hamster groups. In sharp contrast, hamsters without prior vaccination or infection were unprotected and developed the expected hallmarks of COVID-19-like pneumonia, including marked bronchointerstitial pneumonia with necrosuppurative bronchitis and bronchiolitis, diffuse alveolar damage, perivascular and alveolar edema, proliferation of alveolar type II epithelia, and vascular endotheliitis (Figure 6). Of note, the lambertosis-like alveolar bronchiolization, a previously described mid- to long-term complication of COVID-19-like pneumonia in Syrian hamsters,^34^ was observed exclusively in challenge-infected animals that had previously been infected with WT virus.

### Co-infection *in vivo* does not give rise to viral recombinants

**Figure 7:**
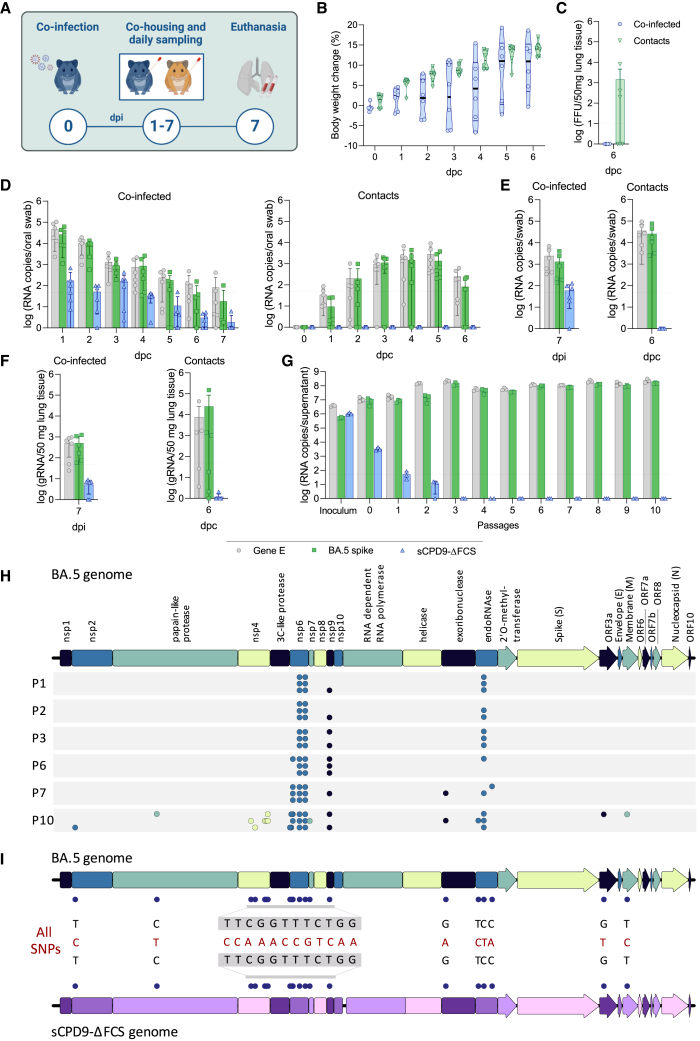
*In vivo* and *in vitro* co-infection with sCPD9-ΔFCS and BA.5 (A) Experimental design. Syrian hamsters were co-infected with sCPD9-ΔFCS and the Omicron variant BA.5. One day post infection (dpi), naive contact hamsters were placed in cohabitation with infected hamsters. Oral swabs were collected daily. All animals were euthanized after 6 days of cohousing. (B) Change in body weight of animals after co-infection or contact to co-infected animals. Violin plots (truncated) show weights of individual animals (n = 6), group medians, and quartiles. (C) Infectious virus particles in homogenized lung tissue. Viral gRNA copies detected in oral swabs (D), oropharyngeal swabs (E), and lung tissue (F) from co-infected and contact hamsters (n = 6), using assays targeting the SARS-CoV-2 E gene (envelope), which is present in both viruses, or sequences that are uniquely present in one of the two different viruses—the spike gene of Omicron BA.5 virus, or the recoded sCPD9 region of sCPD9-ΔFCS virus. (G) Replication of Omicron BA.5 and sCPD9-ΔFCS in CaLu-3 cells. CaLu-3 cells were infected with 1,000 ffu of sCPD9-ΔFCS and 100 ffu of BA.5 and after 72 h of incubation, 1% of the supernatant was used as an inoculum for the next virus passage (n = 3). (C)–(G) Error bars show SD with symbols indicating individual results. Dotted lines represent the limits of detection. (H, I) CaLu-3 cells were infected with 1,000 ffu of sCPD9-ΔFCS and 100 ffu of BA.5 and after 72 h of incubation, either 1% or 5% of the supernatant was used as an inoculum for the next virus passage (n = 3). (H) SNPs identified in passages 1, 2, 3, 6, 7, and 10 of the co-infection experiments and their respective location within the BA.5 reference genome. The panel shows the SNPs identified in the three replicates that contained the most SNPs in each passage, irrespective of the passaging condition. Only SNPs that were identified in >10% of sequence reads are depicted. (I) All unique SNPs that emerged during the co-infection experiments (with >10% read support) in comparison with both BA.5 and sCPD9-ΔFCS genome reference sequences.

To examine potential recombination between vaccine and circulating field virus *in vivo*, Syrian hamsters were infected with equal quantities of sCPD9-ΔFCS and the Omicron variant BA.5 (1 × 10^4^ ffu/animal). After 24 h, infected animals were cohoused with naive contact hamsters to assess host-to-host transmission (Figure 7A). Compared with contacts, infected animals showed a wider distribution of body weights with mild, transient body weight loss in some individuals (Figure 7B). No replication-competent virus was detected in lungs of experimentally infected animals on day 7 after infection (Figure 7C). Low levels of replication-competent virus were detected in lung tissue of three contact hamsters on day 6 of cohabitation. RT-qPCRs performed on daily oral swab samples of infected hamsters showed that sCPD9-ΔFCS was only detectable within the first 3 days after infection, while BA.5 was detected during the entire course of the experiment (Figure 7D). In contrast, minor amounts of sCPD9-specific genomic RNA (gRNA) copies were found in more sensitive oropharyngeal swabs (Figure 7E). No sCPD9-specific gRNA was found in swab and lung samples collected from contact animals, while BA.5-specific RNA was detected abundantly in daily swabs from day 2 post contact, as well as in oropharyngeal swabs and lungs (Figures 7D–7F). This indicates transmission of BA.5 while sCPD9-ΔFCS remained non-transmissible under co-infection conditions. A recombination event that would restore the FCS in the vaccine virus, thereby enabling transmission of vaccine virus, is thus not observed in our experimental setup.

### *In vitro* co-culture does not suggest important recombination events

CaLu-3 cells were infected with 1,000 ffu of sCPD9-ΔFCS and 100 ffu of BA.5. The resulting virus population was passaged 10 times serially on CaLu-3 cells to assess recombination events between the vaccine and field virus. RNA was extracted from cell culture supernatant of each passage. A qPCR assay targeting a conserved region within the SARS-CoV-2 E gene was used to assess total SARS-CoV-2 gRNA content, while assays targeting the FCS region in the spike gene of the BA.5 virus and the genetically recoded sCPD9 region were employed to discriminate between the vaccine and field viruses. Consistent with our *in vivo* data, qPCR results showed that the sCPD9-ΔFCS was outgrown by the BA.5 virus within one passage. The sCPD9-specific RNA levels dropped to levels around the limit of detection by passage 1, while BA.5 maintained replication, resulting in high levels of gRNA over the entire range of the experiment (Figure 7G). This suggests that sCPD9-ΔFCS has a considerable growth disadvantage in cell culture, which limits the possibility of recombination between the vaccine and field virus in the same replication compartments. Consequently, the risk of recombination between sCPD9-ΔFCS and WT viruses is reduced.

To ascertain the absence of high-fitness recombinants, we performed total RNA sequencing of cell culture supernatants from different passages and replicates of co-cultured viruses. Our sequencing analysis showed that, from passage one onward, all sequences above the detection threshold were derived only from the BA.5 virus. We did not find evidence for the presence of sCPD9-ΔFCS-derived sequences in any of our analyses. While some *de novo* mutations appear to have been selected, likely indicating adaptation to cell culture, we can exclude the emergence of sCPD9-ΔFCS/BA.5 recombinants that would have a selective advantage over the BA.5 virus in cultured human cells in our experimental setup (Figures 7H and 7I).

### sCPD9-ΔFCS is safe and immunogenic also for immunosuppressed hamsters

**Figure 8:**
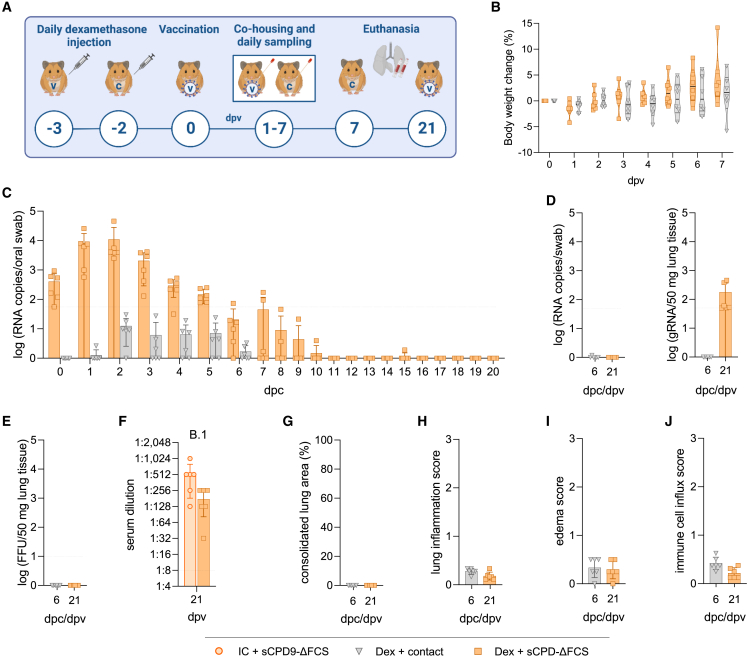
Effect of immunosuppression on safety, efficacy, and transmission of sCPD9-ΔFCS (A) Schematic overview of the experimental setup. Syrian hamsters were immunosuppressed by daily subcutaneous injections of dexamethasone (Dex) at a dose of 2 mg/kg, starting 3 days prior to vaccination or contact. After 3 days of treatment, the hamsters were vaccinated with sCPD9-ΔFCS and subsequently cohoused with naive and immunosuppressed contact animals 24 h after vaccination. Oral swabs were collected daily from all hamsters. Contacts were euthanized 6 days post contact (dpc), while vaccinated hamsters were euthanized 21 days post vaccination (dpv). (B) Change in body weight of immunosuppressed animals after vaccination or contact with vaccinated hamsters. Violin plots (truncated) show weights of individual animals (n = 6), group medians, and quartiles. (C) Viral gRNA copies in oral swabs. (D) gRNA copies in oropharyngeal swabs and lung tissue. (E) Replication-competent virus in lung tissue. (F) Neutralizing activity of sera collected from immunocompetent (IC) and immunosuppressed and sCPD9-ΔFCS-vaccinated animals 21 days post vaccination (dpv) against SARS-CoV-2 variant B.1 (upper detection limit = 1:1,024). A Mann-Whitney test failed to identify siginficant (p < 0.05) differences. (C)–(J) Error bars show SD with symbols representing individual values (n = 6). Dotted lines indicate the limits of detection.

Immunosuppression of Syrian hamsters was induced and maintained by daily administration of dexamethasone (2 mg/kg). Three days after treatment start, animals were vaccinated with sCPD9-ΔFCS, allowing us to determine the effects of immunosuppression on vaccine safety and the humoral immune response to vaccination (Figure 8A). Upon vaccination, immunosuppressed hamsters presented with stable body weights and absence of clinical illness (Figure 8B). SARS-CoV-2 RNA was detectable up to 8 days after vaccination in oral swabs, with highest levels observed on day 1 and 2 (Figure 8C). After 8 days, no viral RNA was detected in oral and oropharyngeal swabs (Figures 8C and 8D). Low levels of gRNA were detected in lung tissue on day 21 after vaccination, suggesting prolonged virus replication in the lower respiratory tract compared with the upper respiratory tract (Figure 8D). However, no replication-competent virus could be recovered from lung samples collected at that time point (Figure 8E). Serum neutralization assays allowed us to determine humoral immune response in immunosuppressed animals 21 days after receiving a single dose of sCPD9-ΔFCS. By trend, serum neutralization titers are lower in immunosuppressed animals compared with immunocompetent animals that received the same vaccination. Nevertheless, despite immunosuppression, all hamsters developed a substantial humoral response to vaccination (Figure 8F). Moreover, lung histopathology was assessed and revealed no evidence of pneumonia in immunosuppressed hamsters after vaccination with sCPD9-ΔFCS (Figures 8G–8J). Overall, sCPD9-ΔFCS remains safe and immunogenic in animals receiving high-dose glucocorticoid treatment.

### sCPD9-ΔFCS is not transmissible between immunosuppressed hamsters

Next, we determined whether the sCPD9-ΔFCS virus was transmissible between immunosuppressed animals. To test this, six immunosuppressed and sCPD9-ΔFCS-vaccinated animals described above were brought into contact with six dexamethasone-treated and immunologically naive hamsters and cohabited for 6 consecutive days (Figure 8A). Throughout the entire cohabitation period, no clinical signs of disease or significant body weight loss were observed (Figure 8B). Although vaccine virus RNA was abundantly detected in oral swabs from vaccinated individuals (Figure 8C), no transmission of vaccine virus was observed, as evidenced by the absence of detectable virus RNA in both upper and lower respiratory tracts of immunosuppressed contact animals (Figures 8D and 8E).

## Discussion

### Removal of FCS has pronounced effects on virus phenotype and prevents transmission

Unlike closely related betacoronaviruses, SARS-CoV-2 can enter the host cell via two distinct entry routes.^21^ If the target cells express TMPRSS2, a protease commonly expressed by epithelial cells of the respiratory, gastrointestinal, and genitourinary tract, the S glycoprotein is efficiently cleaved at the S2′ site, and the virus rapidly enters the cells by fusion with the plasma membrane.^21^ In contrast, if target cells do not produce TMPRSS2 or if the virus lacks a functional FCS, the virus is first internalized into endosomes/lysosomes where the S is cleaved by the cathepsin L or B protease, and enters the cytosol by fusion with the vesicle membrane.^21^

Deletion of the FCS from S has a pleiotropic effect, affecting cell entry, replication, infectivity, and transmissibility of the virus. Experiments in ferrets and Syrian hamsters with SARS-CoV-2 mutants lacking the FCS have shown that the mutants were less pathogenic than WT virus and were shed from the upper respiratory tract of infected animals at lower levels.^25^^,^^26^ In addition, unlike WT virus, the mutant viruses were not transmitted to contact animals.^25^ Thus, efficient spread of SARS-CoV-2 between airway epithelial cells appears to depend on a functional FCS, and priming of S by furin or furin-like proteases.^25^ Acquisition of the FCS can be considered a genetic innovation that provides SARS-CoV-2 with a selective advantage for replication in the respiratory tract of susceptible host species. Conversely, elimination of the FCS reduces the biological fitness of the virus by preventing efficient spread in susceptible host populations. In addition, it has been suggested that SARS-CoV-2 exploits another advantage of entering the cell directly by fusion with the plasma membrane. By this entry mechanism, the virus may escape recognition by the antiviral restriction factors IFITM2 and IFITM3, which are located in the endosome/lysosome.^25^

In our experiments, we observed that the highly attenuated LAV candidate sCPD9 readily spreads to contact animals at levels comparable to the WT virus, albeit without causing disease in either experimentally or naturally infected subjects. Despite the apparent safety of sCPD9, the considerable spread we observed presents a major hurdle for its widespread use as a vaccine. Removal of the FCS thus provides an excellent approach to eliminate transmission and further increase vaccine safety by introducing an independent attenuating mechanism, while maintaining vaccine efficacy.

### sCPD9-ΔFCS is slightly more attenuated than sCPD9, but equally protective

Our results indicate that removal of the FCS from our LAV candidate sCPD9 results in a non-transmissible and highly attenuated virus in the Syrian hamster model. Despite the high level of attenuation, sCPD9-ΔFCS virus showed equal protection against SARS-CoV-2 variant Delta as the parental sCPD9 virus that has a functional FCS. In terms of virological, serological, and histopathological parameters, both the sCPD9 and sCPD9-ΔFCS vaccine viruses provided excellent and similar protection against virus replication and disease upon challenge infection. Overall, protection conferred by either vaccine virus was similar to that induced by infection with WT virus. By trend, animals previously infected with WT virus developed stronger signs of inflammation in the lungs at day 2 after challenge infection. However, this tendency proved to be transient and resolved by day 5 after challenge infection. It is very important and encouraging to note that lambertosis-like alveolar bronchiolization, a previously described mid- to long-term complication of various lung lesions in humans and also COVID-19-like pneumonia in Syrian hamsters^34^ was exclusively observed after previous infection with the WT virus, but not after vaccination with sCPD9 or sCPD9-ΔFCS. While the mechanism and true relevance of this phenomenon are not entirely clear, our observations suggest long-term changes after previous infection with WT virus that were entirely absent in sCPD9- or sCPD9-ΔFCS-vaccinated animals, thus suggesting a significant safety benefit for LAV vaccination compared with infection, even beyond the acute phase of the WT virus-induced disease. Despite multi-level attenuation and complete absence of vaccine virus spread, our results also clearly show that an FCS-lacking LAV retains its full protective potential and exerts protection comparable to previous infection with WT virus. Most importantly, this protection is achieved without any of the negative short- or longer-term effects caused by infection with the WT virus.

### sCPD9-ΔFCS is safe in immunosuppressed animals and shows no recombination with a circulating SARS-CoV-2 variant

Our infection experiments in dexamethasone-treated animals suggest that sCPD9-ΔFCS is also safe for individuals undergoing glucocorticoid treatment. Glucocorticoids are well known for their immunosuppressive effects and are frequently used to treat airway inflammation, making them a specifically relevant immunosuppressive treatment in the context of COVID-19. We previously demonstrated that dexamethasone treatment exerts strong immunosuppressive effects and increases SARS-CoV-2 replication in Syrian hamsters.^35^ Despite this, we found that replication of vaccine virus sCPD9-ΔFCS remained moderate under dexamethasone treatment, with RNA levels decreasing toward the limit of detection within a week following vaccination. Dexamethasone-treated hamsters neither show clinical signs of disease nor any apparent pathology. Moreover, the vaccine virus remained non-transmissible and induced a considerable humoral immune response in dexamethasone-treated animals. These results present a preliminary indication of safety and efficacy of sCPD9-ΔFCS in individuals receiving glucocorticoid treatment.

Both our *in vitro* and *in vivo* co-infection experiments demonstrated that LAV virus sCPD9-ΔFCS is rapidly and consistently outcompeted by the Omicron BA.5 field isolate, indicating a strong selective disadvantage of the attenuated vaccine virus. This limits the replication of vaccine and field viruses in the same host or host compartment, which in turn limits the potential for recombination events between the two viruses. While we cannot formally exclude recombination events between the two viruses in our experimental setup, our sequence analysis allows us to confirm that no recombinant has gained a selective advantage over the BA.5 variant in our co-infection experiments. Furthermore, our *in vivo* co-infection experiments suggest that co-infection with vaccine and field virus does not result in increased virulence, nor does it yield a transmissible vaccine virus. While more work on the topic of recombination is required, our results suggest a limited potential for problematic recombination between the vaccine and circulating field viruses.

Serological data suggest considerable cross-protection conferred by LAV vaccination against different SARS-CoV-2 variants. The broad range of antigens presented by the vaccine, as well as efficient induction of mucosal immunity, are likely causing greater resilience of protection against infection and disease compared with currently available intramuscularly administered and “Spike-only”-based vaccines, specifically in the context of continued SARS-CoV-2 evolution and immune escape.^17^

Similar to LAV, conventional, whole-virus inactivated vaccines, such as CoronaVac (Sinovac Biotech), BIBP-CorV (Sinopharm), Covaxin (Bharat Biotech), or VLA2001 (Valneva) vaccines, present the immune system with the entire antigenic repertoire of the virus. This allows for a broader immune response compared with vaccines that feature only one viral component. However, inactivated virus vaccines, unlike LAV, do not replicate and therefore may not stimulate the immune system to the same extent, particularly in terms of mucosal immunity. While these vaccines have demonstrated efficacy in preventing severe illness, hospitalization, and death, their overall protection level is inferior to that of mRNA vaccines and mostly also to adenoviral-vectored vaccines.^36^^,^^37^^,^^38^^,^^39^ Despite these limitations, inactivated virus vaccines played a vital role in the fight against COVID-19, especially in areas where other types of vaccines were not available or accessible.

### Benefits of FCS removal for mass production of LAV SARS-CoV-2 vaccines

We and others have shown that SARS-CoV-2 variants lacking a functional FCS become rapidly dominant upon passage on TMPRSS2-deficient cells.^24^^,^^27^^,^^28^^,^^29^^,^^30^^,^^31^^,^^32^ These results suggest that mutant viruses lacking functional FCS have a strong selection advantage for replication in cells that do not express TMPRSS2. We here confirm these observations, as the sCPD9-ΔFCS mutant replicated slightly faster than the original sCPD9 virus in Vero E6 cells. Thus, removal of FCS from SARS-CoV-2 LAV has two advantages for potential LAV production. Using non-transgenic Vero cell derivates to produce LAV seems the practically most feasible option. Additionally, faster replication in these cells allows us to efficiently produce large amounts of vaccine doses, reduces production costs, and thus makes LAV a feasible option for efficient and cost-effective mass vaccination. The second major advantage of removing FCS is that it greatly improves the genetic stability of the LAV candidate. One of the prerequisites for the use of LAV in humans is that the vaccine virus population retains a high degree of genetic homogeneity and stability. Since SARS-CoV-2, including our LAV candidate sCPD9, rapidly loses its FCS after passage on Vero cells,^18^ the removal of FCS eliminates the problem of genetic instability at this important site. Importantly, our previous work also indicates high genetic stability within the recoded region of sCPD9.^18^

## Materials and methods

### Cells

Minimal essential medium (MEM) supplemented with 10% fetal bovine serum (FBS), 100 IU/mL penicillin G, and 100 μg/mL streptomycin was used to cultivate Vero E6 (ATCC CRL-1586). For Vero E6-TMPRSS2 cell culture (NIBSC 100978), the medium also contained 1,000 μg/mL geneticin (G418) to select for cells expressing TMPRSS2. CaLu-3 cells were cultivated in DMEM/F12 (1:1) medium containing 20% FBS, 100 IU/mL penicillin G, and 100 μg/mL streptomycin and 1% non-essential amino acids. The cells were kept at 37°C and 5% CO_2_.

### Viruses

The SARS-CoV-2 variants B.1 (B.1, hCoV-19/Germany/BY-ChVir-929/2020, EPI_ISL_406862), Delta (B.1.617.2, Human, 2021, Germany ex India, 20A/452R, EVAg: 009V-04187), and the SARS-CoV-2 mutants B.1-ΔFCS, sCPD9, and sCPD9-ΔFCS were cultured on Vero E6-TMPRSS2 cells. The SARS-CoV-2 variants Omicron BA.1 (BA.1.18, hCoV-19/Germany/BE-ChVir26335/2021, EPI_ISL_7019047) and BA.5 (BE.1.1, hCoV-19/Germany/SH-ChVir29057_V34/2022, EPI_ISL_16221625) were propagated on CaLu-3 cells. The BAC-derived SARS-CoV-2 variant B.1 (GenBank: MT108784) was grown on Vero E6 cells and used for growth kinetics and plaque size assays. Titers of virus stocks were determined by conducting plaque assays on Vero E6 cells, and the viruses were stored at −80°C.

### Ethics statement

*In vivo* and *in vitro* experiments were carried out at the Institut für Virologie, Freie Universität, Berlin, Germany, in a biosafety-level three (BSL-3) laboratory. The animal experiments were conducted according to institutional, national, and international guidelines for the care and ethical use of animals, and were authorized by the competent local authority, Landesamt für Gesundheit und Soziales, Berlin (permit number 0086/20).

### Study design and animal husbandry

The objective of this study was to determine the impact of removing the FCS from the LAV candidate sCPD9 on its immunogenicity, protective efficacy, and host-to-host transmission. The transmissibility of sCPD9 virus lacking the FCS (sCPD9-ΔFCS) was compared with that of the WT SARS-CoV-2 variant B.1 and sCPD9 viruses. Additionally, potential recombination between SARS-CoV-2 variant Omicron BA.5 and the vaccine candidate sCPD9-ΔFCS virus was assessed in a co-infection experiment. Furthermore, the safety and transmissibility of the sCPD9-ΔFCS vaccine candidate were investigated in immunosuppressed hamsters.

Syrian hamsters (*Mesocricetus auratus*; breed RjHan:AURA) were purchased from Janvier Labs and housed in pairs in individually ventilated cages. Food and water were provided *ad libitum*, and cages were enriched with nesting material. The room temperature was maintained at a constant range of 22–24°C with a relative humidity of 40%–55%. Prior to the beginning of the experiment, the animals were allowed to acclimate to the housing conditions for 7 days.

The transmission of B.1, sCPD9, and sCPD9-ΔFCS viruses from infected to naive animals was evaluated using 36 male and female Syrian hamsters that were 10 weeks old. After acclimation, one animal from each cage was infected with one of the three viruses by intranasal instillation under general anesthesia (0.15 mg/kg medetomidine, 2.0 mg/kg midazolam, and butorphanol 2.5 mg/kg). A total of six animals each were infected with 1 × 10^5^ ffu B.1, or vaccinated with 1 × 10^5^ ffu sCPD9 or sCPD9-ΔFCS viruses in 60 μL MEM. To prevent accidental transmission of the virus from the inoculum to naive contact animals, infected/vaccinated hamsters were quarantined in separate cages for 24 h and then reunited with their uninfected partners.

Body weights of all hamsters were recorded daily, and clinical conditions were assessed twice daily. Oral swabs were taken daily from contact hamsters on days 1–6 post contact (dpc). Contact animals were euthanized at 6 dpc to assess viral loads in upper and lower respiratory tract, signs of pneumonia, and seroconversion.

sCPD9-, sCPD9-ΔFCS-, and B.1-infected animals were challenge-infected with 1 × 10^5^ ffu of the SARS-CoV-2 Delta variant on day 21 after infection. In addition, a group of six uninfected hamsters was challenged with the Delta variant for control purposes. On days 2 and 5 post challenge (dpch), three hamsters per group were euthanized, and blood, trachea, and lungs were collected for virological, serological, and histopathological analyses.

To investigate the potential for recombination between SARS-CoV-2 variant Omicron BA.5 and sCPD9-ΔFCS, a co-infection experiment was performed using 12 female Syrian hamsters that were 4 weeks old. After acclimation, six hamsters were co-infected with 1 × 10^4^ ffu of sCPD9-ΔFCS and 1 × 10^4^ ffu of SARS-CoV-2 variant Omicron BA.5 by intranasal instillation. The infection was conducted under general anesthesia, which was induced as described above. Subsequently, the infected animals were quarantined in separate cages to prevent accidental transmission of the virus inoculum to naive animals. After 1 day, they were again cohoused with their naive partners. Body weights and oral swabs were collected daily from all animals to screen for virus transmission. Clinical conditions were monitored twice daily. After 6 days of cohousing, animals were euthanized to collect blood, trachea, and lungs for virological analyses.

To assess vaccine safety and transmissibility in immunosuppressed animals, 12 female Syrian hamsters that were 4 weeks old were immunocompromised by daily subcutaneous injection of dexamethasone (2 mg/kg). On the third day of immunosuppression, six hamsters were vaccinated 1 × 10^4^ ffu of sCPD9-ΔFCS by intranasal application performed under general anesthesia as described above. The vaccinated animals were housed in individual cages for 24 h and subsequently reunited with their unvaccinated and immunosuppressed conspecifics. During the cohousing period, oral swabs were taken daily from all hamsters to detect potential vaccine virus transmission. The contact animals were euthanized at 6 dpc, while vaccinated hamsters were euthanized 21 days post vaccination (dpv). Blood, trachea, and lungs were collected for serological, virological, and histopathological analyses.

### Virus growth kinetics

T25 flasks containing confluent Vero E6 or Vero E6-TMPRSS2 cells were infected with 100 ffu of sCPD9-ΔFCS, sCPD9, B.1-ΔFCS, or B.1. Virus was diluted in 5 mL of complete cell culture medium and added to each flask. At 24, 48, 72, and 96 h after infection, virus was released from infected cells by a freeze-thaw cycle and virus titers were determined by plaque assay on confluent Vero E6 cells in 12-well plates. For this purpose, cells were infected with 10-fold virus dilutions for 75 min, overlaid with MEM medium containing 1.5% carboxymethylcellulose sodium (Sigma Aldrich), and incubated for 48 h. Cells were then fixed with 4% PBS-buffered formaldehyde (pH 6.5) and plaques were visualized by immunofluorescence staining.^11^

### Co-culture of sCPD9-ΔFCS and Omicron BA.5

CaLu-3 cells were seeded in T25 flasks and grown to a density of 90%. Prior to co-infection with 100 ffu of SARS-CoV-2 variant Omicron BA.5 and 1,000 ffu of sCPD9-ΔFCS, cell culture medium was changed to 5 mL DMEM/F12 1:1 containing 10% FBS, 100 IU/mL penicillin G, 100 μg/mL streptomycin, and 1% non-essential amino acids. After 72 h, the supernatant was harvested and cleared by centrifugation at 5,000 rpm for 15 min. Subsequently, 1% of supernatant was transferred to previously uninfected CaLu-3 cells. The assay was performed in triplicate and continued for a total of 10 passages.

### RNA extraction and RT-qPCR

SARS-CoV-2 RNA was detected in oral, oropharyngeal swabs, lung tissue, and cell culture supernatant by reverse transcription quantitative PCR (RT-qPCR). RNA was isolated from swab and lung samples using the innuPREP Virus DNA/RNA Kit (Analytik Jena). Prior to RNA isolation, 2.5 mg of lung tissue was homogenized in a bead mill (Analytik Jena). RNA was extracted from 250 μL of cell culture supernatant of the co-culture experiment using Trizol LS Reagent (Thermo Fisher Scientific).

SARS-CoV-2 RNA was quantified with the NEB Luna Universal Probe One-Step RT-qPCR Kit (New England Biolabs) using an assay targeting the SARS-CoV-2 envelope (*E*) gene for all experiments.^40^ To quantify SARS-CoV-2 variant BA.5 in samples from co-cultivation trials, we used a BA.5-specific primer and probe set targeting the FCS region of the spike gene (forward primer: 5′-TCAGACTAAGTCTCATCGG-3′, reverse primer: 5′-CTGATGTCTTGGTCATAGACAC-3′, probe: 5′-FAM-TGCTTACTCTAATAACTCTATTGCCATACCCAC-BHQ1-3′). To detect the sCPD9 and sCPD9-ΔFCS viruses, we used primers targeting the recoded sCPD9 region (forward primer: 5′- TCCGTTGCGATTAAGATTACC-3′, reverse primer: 5′- GAACTAGAAGCGTTAACATTCG-3′, probe: 5′-FAM- TCATTTCGCATGGTGGACTGCATTC-BHQ1-3′). qPCR was performed on a qTower G3 cycler (Analytik Jena) using the following cycling conditions: 10 min at 55°C for reverse transcription, 3 min at 94°C for activation of the enzyme, and 40 cycles of 15 s at 94°C and 30 s at 58°C.

### Plaque assay

To quantify replication-competent virus, 10-fold serial dilutions of 50 mg homogenized lung tissue were prepared in MEM and plated on Vero E6 cells grown in 12-well plates. After incubation for 2.5 h at 37°C and 5% CO_2_, cells were overlaid with 1.5% carboxymethylcellulose sodium (Sigma Aldrich) diluted in complete growth medium. Cells were fixed 72 h after infection with 4% PBS-buffered formaldehyde (pH 6.5). Cells were stained with 0.75% methylene blue and plaques were counted.

To measure plaque sizes, 100 ffu of virus was used to infect Vero E6 cells grown in a single well of a 12-well plate. Forty-eight hours after infection, cells were fixed with 4% PBS-buffered formaldehyde (pH 6.5) and infected cells were stained with fluorescently labeled antibodies.^11^ To determine plaque sizes, images of 50 randomly selected plaques were taken at 50-fold magnification using an inverted fluorescence microscope Axiovert S100 (Zeiss). The plaque areas were measured using ImageJ software,^41^ from which the plaque diameters were calculated.

### Neutralization test

Serum samples from hamsters of the first transmission experiment, as well as vaccinated and immunosuppressed hamsters, were tested for neutralizing capacity against SARS-CoV-2 variant B.1. Additionally, sera from challenge-infected animals at 0, 2, and 5 dpch were tested for neutralizing activity against SARS-CoV-2 Delta variant (B.1.617) and the Omicron variants BA.1 and BA.5. Due to lack of material, sera collected on day 0 could not be tested for neutralizing antibodies against BA.5. To this end, sera were inactivated at 56°C for 30 min. Two-fold serial dilutions ranging from 1:8 to 1:1,024 were prepared in 96-well plates, and 200 ffu of SARS-CoV-2 diluted in MEM (1% FBS, 1% P/S) were added to all wells. After incubation at 37°C for 1 h, the dilutions were added to subconfluent Vero E6 cells grown in 96-well cell culture plates. The cells were then incubated for 72 h, and then fixed with 4% formaldehyde and stained with aqueous 0.75% methylene blue solution. Any wells that showed no virus-induced cytopathic effect were considered neutralized and the corresponding serum titer was reported. Each plate included positive and negative controls to ensure accuracy of the results.

### Histopathology

After euthanasia, the left lung lobe was removed and immersed in a 4% PBS-buffered formaldehyde solution for 48 h to fix the tissue. After fixation, lung tissues were embedded in paraffin and cut into 2-μm-thick slices, which were then stained with hematoxylin and eosin. The severity of pneumonia and other histological changes were scored using a standardized procedure, as described previously.^42^

### Sequencing

Following RNA extraction from cell culture supernatant as described above, libraries were prepared and sequenced using Illumina technology (Illumina). For library preparation, the NEBNext Ultra II RNA Library Prep Kit for Illumina (New England Biolabs) was used. This approach relies on standard library preparation steps for Illumina sequencing, such as end repair, adaptor ligation, and PCR enrichment. Quantification of enriched sequencing libraries was performed using the NEBNext Library Quant Kit for Illumina (New England Biolabs). Libraries were then pooled and sequenced on an Illumina Miseq System (Illumina). All raw generated sequencing data are available at the SRA (Sequencing Read Archive) under BioProject ID PRJNA951657.

The generated Illumina sequencing data were processed with Trimmomatic v.0.39^43^ and mapped against BA.5 (NCBI accession number: ON249995) and sCPD9-ΔFCS genome references (GenBank: MZ064545.1),^11^ respectively, using the Burrows-Wheeler aligner v.0.7.17.^44^ Mapping statistics were generated using Samtools v1.10^45^ and alignments were visualized using IGV v2.9.4 for Linux.^46^ For detection of single nucleotide polymorphisms (SNPs), Freebayes, a Bayesian genetic variant detector (arXiv:1207.3907 [q-bio.GN] 2012) was used. All SNPs with a minimum mapping quality of 5, minimum count of 3, and minimum fraction of 0.1 were initially considered. SNPs detected between the starting BA.5 isolate used in these studies and the BA.5 reference were removed from further analysis, as they were present before these experiments. A table containing the removed SNPs is provided. Consensus sequences for each sample were obtained using BCFtools.^45^

Direct sequence comparison to sCPD9-ΔFCS is not efficient given the slight differences in genomic structure between BA.5 and sCPD9-ΔFCS and high entropy between the samples and the sCPD9-ΔFCS reference. To facilitate this process, a sample-wide consensus sequence containing all detected SNPs was created on a backbone of the BA.5 reference (the most similar to all samples) using SNP-sites^47^ and BCFtools. This sample-wide consensus was aligned to both BA.5 and sCPD9-ΔFCS references using EMBOSS Stretcher,^48^ and new SNP tables were created using SNP-sites, to verify location and identity of all detected SNPs against both references. All SNPs occurring between the BA.5 starting isolate and sCPD9-ΔFCS references, and thus not arising during the co-infection experiments, were removed from analysis. Base SNP differences between our BA.5 isolate and reference ON249995, as well as SNP counts for every sample (in relation to the BA.5 and sCPD9-ΔFCS references) and custom references/consensus used or generated during analysis can be found at https://github.com/mmnascimento/scpd9dFCS.

## Data availability

The complete set of raw generated sequencing data can be found at the SRA (Sequencing Read Archive) under BioProject ID PRJNA951657. Base SNP differences between our BA.5 isolate and reference ON249995, as well as SNP counts for every sample and custom references/consensus used or generated during analysis can be found at https://github.com/mmnascimento/scpd9dFCS. All remaining data shown in this work are available from the authors upon request.
